# Supplementary material for: Apelin inhibition prevents resistance and metastasis associated with anti‐angiogenic therapy
Source: EMBO Mol Med. 2019 Jun 24;11(8):e9266. doi: 10.15252/emmm.201809266 (PMC6685079; doi:10.15252/emmm.201809266)
Supplement: Supplementary file 1 — Appendix [file EMMM-11-e9266-s001.pdf]

Appendix

1.) Appendix Figures S1-S3

2.) Appendix Figure Legends S1-S3

3.) Appendix Table S1

Appendix Figure S1

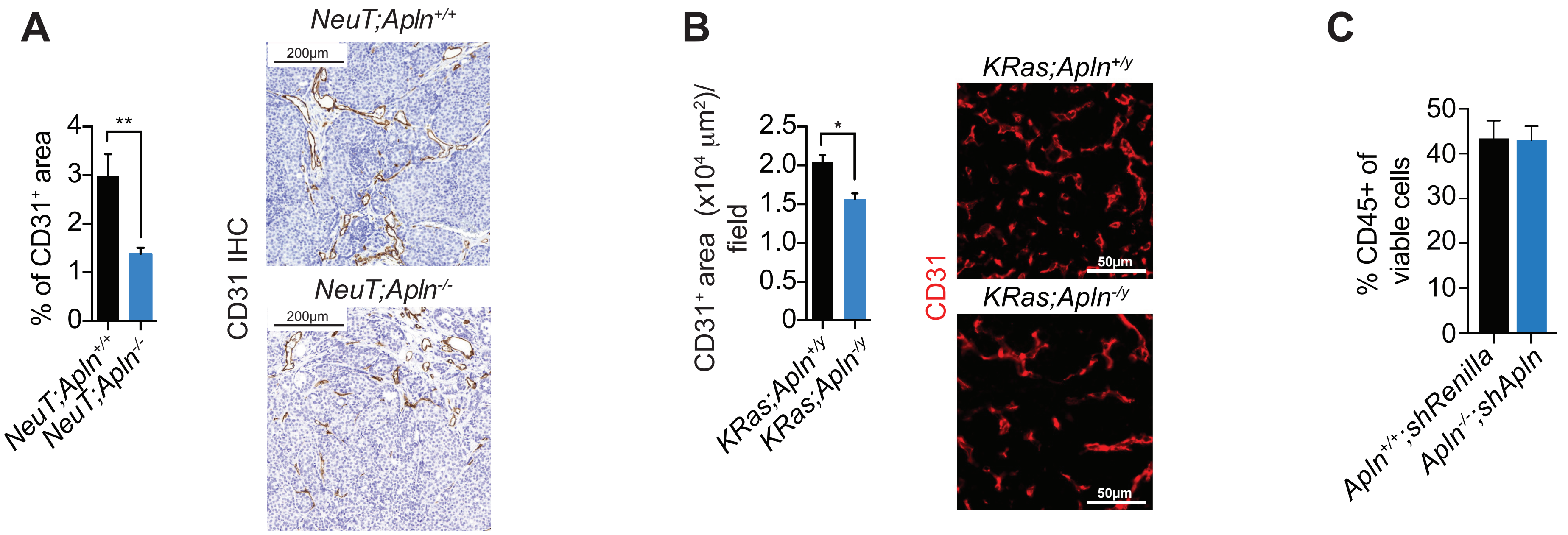

Appendix Figure S2

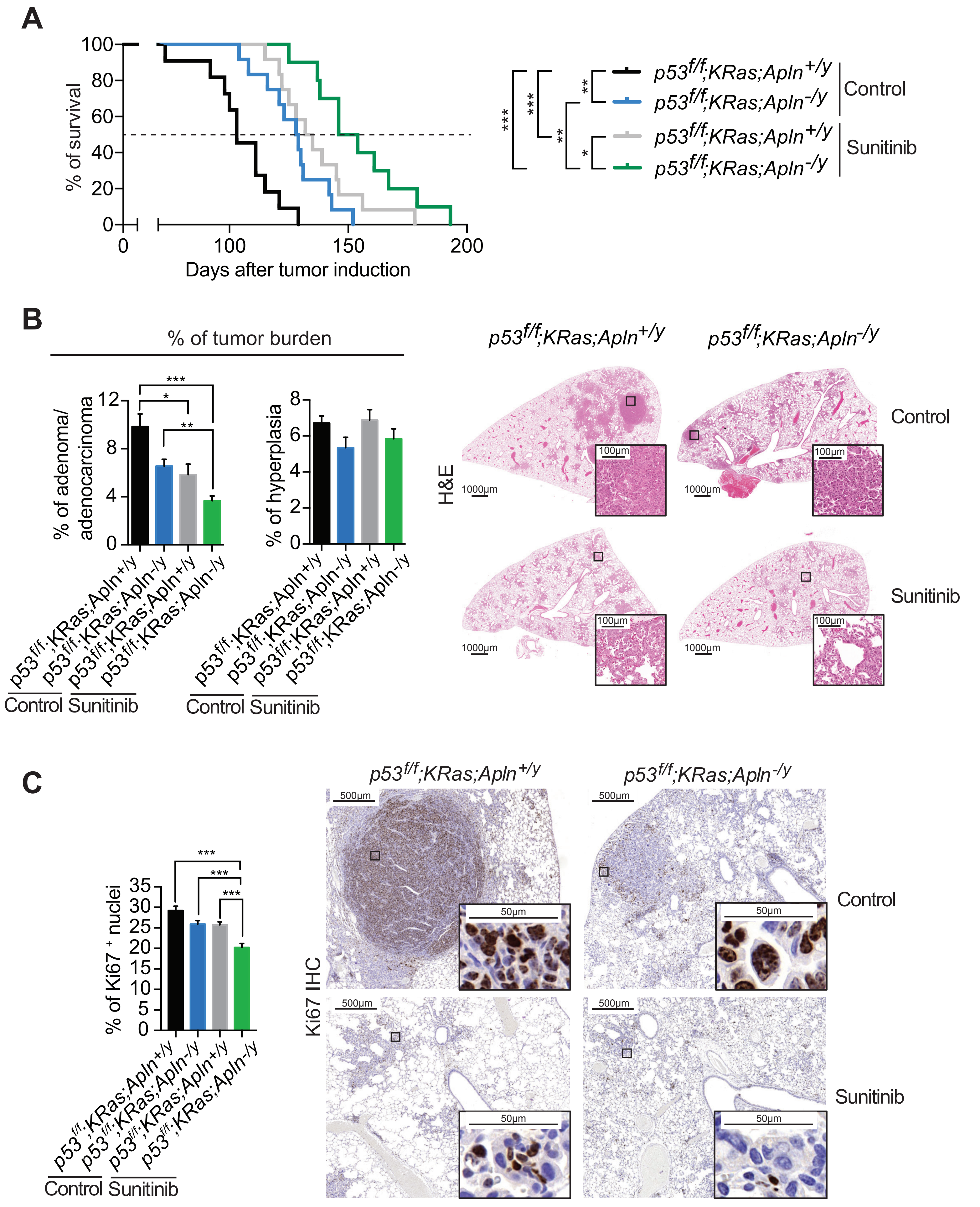

Appendix Figure S3

A

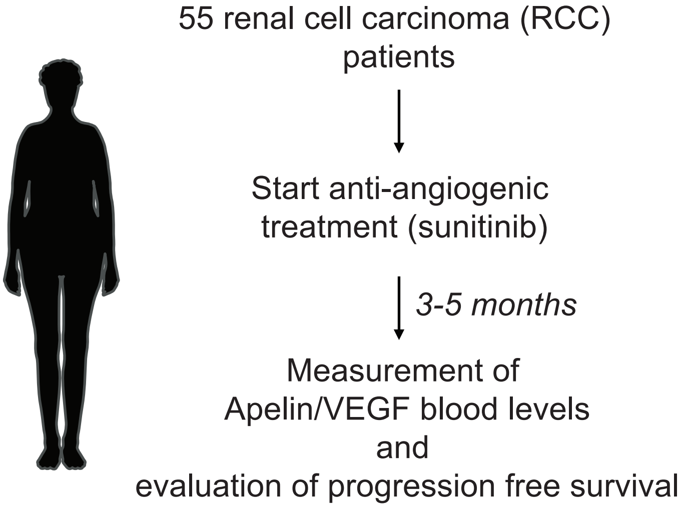

B

Cox regression model			
**Characteristics**	**Exp(B)**	**95% CI**	**P-value**
Age in years (<60 vs ≥60)	0.680	0.383-1.208	0.188
Gender (male vs female)	1.171	0.558-2.456	0.676
T1+T2 vs T3+T4	0.544	0.302-0.981	0.043
Serum APLN level (low vs high)	1.665	0.849-3.263	0.138
**Abbreviations:** Exp(B), odds ratio; CI, confidence interval			

Appendix Figure Legends S1-S3

Appendix S1 – Targeting Apelin reduces angiogenesis in mammary and lung cancer.

(A) Mean percentages of $CD31^+$ area (± S.E.M.) in *NeuT;*$Apln^{+/+}$ (n=3) and *NeuT;*$Apln^{-/-}$ (n=6) mice, 4 weeks after mammary tumor onset. **P<0.01; t test. Right panels show representative immunohistochemical anti-CD31 staining. Scale bars = 200 µm. **(B)** CD31-positive areas (x10^4 µm^2)/field in lung tumors of *KRas;*$Apln^{+/y}$ (n=4) and *KRas;*$Apln^{-/y}$ (n=4) mice 18 weeks after AdenoCre inhalation. *P<0.05; t test; three sections per lung were analysed. Right panels show representative anti-CD31 staining. Scale bars = 50 µm. **(C)** Mean percentage (± S.E.M.) of $CD45^+$ tumor-infiltrating immune cells normalized to viable cell count, evaluated by FACS from digested mammary tumors. E0771 *shRenilla* (n=8) or *shApln* (n=6) were orthotopically injected into C57BL/6J $Apln^{+/+}$ or $Apln^{-/-}$ mice, respectively and tumors were harvested at day 25 post-injection.

Appendix S2 – Apelin-depletion in combination with sunitinib treatment mitigates KRas-driven lung cancer.

(A) Kaplan Meier survival plot of $p53^{f/f}$*;KRas;*$Apln^{+/y}$ and $p53^{f/f}$*;KRas;*$Apln^{-/y}$ mice with non-small lung cancer (NSCLC), either left untreated (control) or treated with sunitinib (60mg/kg, three times per week) after tumor onset. $p53^{f/f}$*;KRas;*$Apln^{+/y}$ Control (n=11), $p53^{f/f}$*;KRas;*$Apln^{-/y}$ Control (n=12), $p53^{f/f}$*;KRas;*$Apln^{+/y}$ Sunitinib (n=12), $p53^{f/f}$*;KRas;*$Apln^{-/y}$ Sunitinib (n=10); *P<0.05; **P<0.01; ***P<0.001; Log rank test. The dotted line indicates 50% survival. **(B)** Percentages of adenoma/adenocarcinoma and hyperplasia in age-matched $p53^{f/f}$*;KRas;*$Apln^{+/y}$ and $p53^{f/f}$*;KRas;*$Apln^{-/y}$ lungs 8 weeks after adenoCre inhalation, either left untreated (control) or treated with sunitinib (60mg/kg, three times per week). Data are shown as mean values ± S.E.M. $p53^{f/f}$*;KRas;*$Apln^{+/y}$ Control (n=9) and $p53^{f/f}$*;KRas;*$Apln^{-/y}$ Control (n=7), $p53^{f/f}$*;KRas;*$Apln^{+/y}$ Sunitinib (n=7) and $p53^{f/f}$*;KRas;*$Apln^{-/y}$ Sunitinib (n=7); three sections per lung were analysed; *P<0.05; **P<0.01; ***P<0.001; Kruskal-Wallis test. Right panels show representative H&E

images. Insets show higher magnifications of lung tumors. Scale bars = 1000 µm (large panels) and 100 µm (insets). **(C)** Ki67 immunohistochemistry of age-matched *p53*$^{f/f}$*;KRas;Apln*$^{+/y}$ and *p53*$^{f/f}$*;KRas;Apln*$^{-/y}$ lungs tumors 8 weeks after adenoCre inhalation, left untreated (control) or treated with sunitinib (60mg/kg, three times per week). Data are shown as mean values ± S.E.M. *p53*$^{f/f}$*;KRas;Apln*$^{+/y}$ Control (n=8), *p53*$^{f/f}$*;KRas;Apln*$^{-/y}$ Control (n=8), *p53*$^{f/f}$*;KRas;Apln*$^{+/y}$ Sunitinib (n=9) and *p53*$^{f/f}$*;KRas;Apln*$^{-/y}$ Sunitinib (n=7); three sections per lung were analysed; ***P<0.001; Kruskal-Wallis test. Right panels show representative Ki67 intra-tumoral stainings. Scale bars = 500 µm (large panels) and 50 µm (insets).

Appendix S3 - Multivariate analysis of patients treated with sunitinib therapy.

(A) Experimental set up for clinical study in RCC (renal cell carcinoma) patients and non-small cell lung cancer patients that received sunitinib or bevacizumab anti-angiogenic therapy, respectively. **(B)** Multivariate analysis of data from RCC patients 3-5 months after the start date of sunitinib treatment; complementary to data in Figure 7C.

Appendix Table S1		
**Figure**	**Comparison**	**p-value**
Figure 1A	NeuT;Apln$^{+/+}$ vs. NeuT;Apln$^{-/-}$	0.0185
Figure 1B	Apln+/+ shRen vs. Apln-/- shApln; Day 17	<0.0001
	Apln+/+ shRen vs. Apln+/+ shApln; Day 20	0.0081
	Apln+/+ shRen vs. Apln-/- shRen; Day 20	0.0076
	Apln+/+ shRen vs. Apln-/- shApln; Day 20	<0.0001
	Apln+/+ shApln vs. Apln-/- shApln; Day 20	0.0004
	Apln-/- shRen vs. Apln-/- shApln; Day 20	0.0018
Figure 1C	Apln+/+ shRen vs. Apln-/- shApln	0.0040
Figure 1D	Apln+/+ shRen vs. Apln-/- shApln	0.0013
Figure 1E	Apln+/+ shRen vs. Apln+/+ shApln	0.0379
Figure 1F	Apln+/+ shRen vs. Apln-/- shApln; PMN-MDSC	0.0015
	Apln+/+ shRen vs. Apln-/- shApln; NKT	0.0234
Figure 2B	Apln GO vs. Apln STOP; Day 6	0.0032
	Apln GO vs. Apln STOP; Day 8	<0.0001
	Apln GO vs. Apln STOP; Day 10	<0.0001
	Apln GO vs. Apln STOP; Day 11	<0.0001
Figure 3A	NeuT;Apln+/+ Ctrl vs. NeuT;Apln+/+ Sut	0.0158
	NeuT;Apln+/+ Ctrl vs. NeuT;Apln-/- Ctrl	0.0153
	NeuT;Apln+/+ Ctrl vs. NeuT;Apln-/- Sut	0.0001
	NeuT;Apln-/- Ctrl vs. NeuT;Apln-/- Sut	0.0339
	NeuT;Apln+/+ Sut vs. NeuT;Apln-/- Sut	0.0251
Figure 3B	NeuT;Apln+/+ Ctrl vs. NeuT;Apln-/- Ctrl	0.0112
	NeuT;Apln+/+ Sut vs. NeuT;Apln-/- Sut	0.0476
	NeuT;Apln+/+ Ctrl vs. NeuT;Apln-/- Sut	0.0002
Figure 3D	NeuT;Apln-/- Ctrl vs. NeuT;Apln-/- Sut	0.0431
	NeuT;Apln+/+ Sut vs. NeuT;Apln-/- Sut	0.0102
Figure 3E	Control vs. MM54; Day 18	0.0120
	Control vs. Sunitinib; Day 18	0.0374
	Control vs. Sunitinib+MM54; Day 18	0.0019
	Control vs. MM54; Day 21	0.0002
	Control vs. Sunitinib; Day 21	<0.0001
	Control vs. Sunitinib+MM54; Day 21	<0.0001
	MM54 vs. Sunitinib+MM54; Day 25	0.0011
	Sunitinib vs. Sunitinib+MM54; Day 25	0.0337
Figure 4C	NeuT;Apln+/+ Ctrl vs. NeuT;Apln-/- Sut; 2 weeks	0.0089
	NeuT;Apln+/+ Ctrl vs. NeuT;Apln+/+ Sut; 4 weeks	0.0056
	NeuT;Apln+/+ Ctrl vs. NeuT;Apln-/- Sut; 4 weeks	0.0007
	NeuT;Apln-/- Ctrl vs. NeuT;Apln-/- Sut; 2 weeks	0.0217
Figure 4D	NeuT;Apln+/+ Ctrl vs. NeuT;Apln+/+ Sut; CD31	0.0043
	NeuT;Apln+/+ Ctrl vs. NeuT;Apln-/- Sut; CD31	<0.0001
	NeuT;Apln-/- Ctrl vs. NeuT;Apln-/- Sut; CD31	0.0439
	NeuT;Apln+/+ Ctrl vs. NeuT;Apln-/- Sut; Dil. Vessel	0.0100

	NeuT;Apln-/- Ctrl vs. NeuT;Apln-/- Sut; Dil. Vessel	0.0034
	NeuT;Apln+/+ Ctrl vs. NeuT;Apln+/+ Sut; CD31/α-SMA	<0.0001
	NeuT;Apln+/+ Ctrl vs. NeuT;Apln-/- Ctrl; CD31/α-SMA	<0.0001
	NeuT;Apln+/+ Ctrl vs. NeuT;Apln-/- Sut; CD31/α-SMA	<0.0001
	NeuT;Apln-/- Ctrl vs. NeuT;Apln+/+ Sut; CD31/α-SMA	<0.0001
	NeuT;Apln-/- Ctrl vs. NeuT;Apln-/- Sut; CD31/α-SMA	<0.0001
	NeuT;Apln+/+ Sut vs. NeuT;Apln-/- Sut; CD31/α-SMA	<0.0001
Figure 5A	NeuT;Apln+/+ Ctrl vs. NeuT;Apln+/+ Sut	<0.0001
	NeuT;Apln+/+ Ctrl vs. NeuT;Apln-/- Sut	<0.0001
	NeuT;Apln-/- Ctrl vs. NeuT;Apln+/+ Sut	<0.0001
	NeuT;Apln-/- Ctrl vs. NeuT;Apln-/- Sut	<0.0001
	NeuT;Apln+/+ Sut vs. NeuT;Apln-/- Sut	<0.0001
Figure 5C	NeuT;Apln+/+ Ctrl vs. NeuT;Apln-/- Ctrl; 4 weeks	<0.0001
	NeuT;Apln+/+ Ctrl vs. NeuT;Apln-/- Sut; 4 weeks	<0.0001
	NeuT;Apln+/+ Sut vs. NeuT;Apln-/- Ctrl; 4 weeks	0.0351
	NeuT;Apln+/+ Sut vs. NeuT;Apln-/- Sut; 2 weeks	0.0437
	NeuT;Apln+/+ Sut vs. NeuT;Apln-/- Sut; 4 weeks	0.0479
Figure 6A	NeuT;Apln+/+ Ctrl vs. NeuT;Apln+/+ Sut	0.0006
	NeuT;Apln+/+ Sut vs. NeuT;Apln-/- Ctrl	0.0101
	NeuT;Apln+/+ Sut vs. NeuT;Apln-/- Sut	0.0011
Figure 6B	Low APLN vs. high APLN	0.0099
Figure 6C	Low APLN vs. high APLN	0.0367
Figure 6D	Low APLN/ Low VEGF vs. Low APLN/High VEGF	0.0356
	Low APLN/ Low VEGF vs. High APLN/Low VEGF	0.0085
	Low APLN/ Low VEGF vs. High APLN/High VEGF	0.0070
Figure EV1A	Low APLN vs. high APLN	0.005
Figure EV1C	NeuT;Apln+/+ vs. NeuT;Apln-/-	0.0019
Figure EV1D	NeuT;Apln+/+ vs. NeuT;Apln-/-	0.00235
Figure EV1E	Low APLN vs. high APLN	0.0019
Figure EV1F	KRas;Apln+/y vs. KRas;Apln-/y	0.0037
Figure EV1G	p53f/f;KRas;Apln+/y vs. p53f/f;KRas;Apln-/y	0.0035
Figure EV1H	KRas;Apln+/y vs. KRas;Apln-/y; Adenoma	0.0117
	KRas;Apln+/y vs. KRas;Apln-/y; Hyperplasia	0.2156
Figure EV2A	NeuT;Apln+/+ mamm tumor vs. Apln+/+ mamm. Gland	0.0017
Figure EV2B	E0771 shRenilla vs. E0771 shApln; Apln mRNA	0.0035
	E0771 shRenilla vs. E0771 shAplnr; Aplnr mRNA	0.005
Figure EV2E	E0771 shApln vs. E0771 shAplnr; Day 18	0.0269
	E0771 shRen vs. E0771 shApln; Day 22	0.0004
	E0771 shApln vs. E0771 shAplnr; Day 22	<0.0001
Figure EV2F	E0771 shRen vs. E0771 shApln	0.0087
	E0771 shApln vs. E0771 shAplnr	0.0351
Figure EV4A	Apln+/+ shRen Ctrl vs. Apln-/- shApln Ctrl; Day 25	0.0006
	Apln+/+ shRen Ctrl vs. Apln+/+ shRen Sut; Day 25	<0.0001
	Apln+/+ shRen Ctrl vs. Apln-/- shApln Sut; Day 25	<0.0001

	Apln+/+ shRen Ctrl vs. Apln-/- shApln Ctrl; Day 28	<0.0001
	Apln+/+ shRen Ctrl vs. Apln+/+ shRen Sut; Day 28	<0.0001
	Apln+/+ shRen Ctrl vs. Apln-/- shApln Sut; Day 28	<0.0001
	Apln-/- shApln Ctrl vs. Apln-/- shApln Sut; Day 28	0.0014
	Apln-/- shApln Ctrl vs. Apln-/- shApln Sut; Day 32	0.0124
	Apln-/- shApln Ctrl vs. Apln+/+ shRen Sut; Day 36	0.0064
	Apln-/- shApln Ctrl vs. Apln-/- shApln Sut; Day 36	0.0002
	Apln+/+ shRen Sut vs. Apln-/- shApln Sut; Day 43	0.0479
Figure EV4B	Apln+/+ shRen Ctrl vs. Apln-/- shApln Axitinib; Day 14	0.0110
	Apln+/+ shRen Ctrl vs. Apln+/+ shRen Axitinib; Day 18	0.0025
	Apln+/+ shRen Ctrl vs. Apln-/- shApln Axitinib; Day 18	<0.0001
	Apln+/+ shRen Ctrl vs. Apln-/- shApln Ctrl; Day 21	0.0004
	Apln+/+ shRen Ctrl vs. Apln+/+ shRen Axitinib; Day 21	<0.0001
	Apln+/+ shRen Ctrl vs. Apln-/- shApln Axitinib; Day 21	<0.0001
	Apln-/- shApln Ctrl vs. Apln-/- shApln Axitinib; Day 21	0.0001
	Apln+/+ shRen Axitinib vs. Apln-/- shApln Axitinib; Day 21	0.0094
Figure EV4C	Apln+/+ shRen Ctrl vs. Apln-/- shApln a-VEGF; Day 19	0.0091
	Apln+/+ shRen Ctrl vs. Apln+/+ shRen a-VEGF; Day 22	0.0055
	Apln-/- shApln Ctrl vs. Apln-/- shApln a-VEGF; Day 22	0.0047
	Apln+/+ shRen Ctrl vs. Apln-/- shApln a-VEGF; Day 22	<0.0001
Figure EV4D	NeuT;Apln+/+ Ctrl vs. NeuT;Apln-/- Sut	0.0023
Figure EV5A	NeuT;Apln+/+ Ctrl vs. NeuT;Apln+/+ Sut	<0.0001
	NeuT;Apln-/- Ctrl vs. NeuT;Apln+/+ Sut	<0.0001
	NeuT;Apln+/+ Sut vs. NeuT;Apln-/- Sut	0.0001
Figure EV5B	E0771 shRen Ctrl vs. E0771 shRen Sut	<0.0001
	E0771 shApln Ctrl vs. E0771 shRen Sut	<0.0001
	E0771 shRen Sut vs. E0771 shApln Sut	<0.0001
Figure EV5C	Apln+/+ shRen Ctrl vs. Apln+/+ shRen Sut	<0.0001
	Apln-/- shApln Ctrl vs. Apln+/+ shRen Sut	<0.0001
	Apln+/+ shRen Sut vs. Apln-/- shApln Sut	<0.0001
Figure S1A	NeuT;Apln+/+ vs. NeuT;Apln-/-	0.0027
Figure S1B	KRas;Apln+/y vs. KRas;Apln-/y	0.0143
Figure S2A	p53f/f;KRas;Apln+/y Ctrl vs. p53f/f;KRas;Apln-/y Ctrl	0.0010
	p53f/f;KRas;Apln+/y Ctrl vs. p53f/f;KRas;Apln+/y Sut	<0.0001
	p53f/f;KRas;Apln+/y Ctrl vs. p53f/f;KRas;Apln-/y Sut	<0.0001
	p53f/f;KRas;Apln-/y Ctrl vs. p53f/f;KRas;Apln-/y Sut	0.0011
	p53f/f;KRas;Apln+/y Sut vs. p53f/f;KRas;Apln-/y Sut	0.0376
Figure S2B	p53f/f;KRas;Apln+/y Ctrl vs. p53f/f;KRas;Apln+/y Sut	0.0190
	p53f/f;KRas;Apln+/y Ctrl vs. p53f/f;KRas;Apln-/y Sut	<0.0001
	p53f/f;KRas;Apln-/y Ctrl vs. p53f/f;KRas;Apln-/y Sut	0.0070
Figure S2C	p53f/f;KRas;Apln+/y Ctrl vs. p53f/f;KRas;Apln-/y Sut	<0.0001
	p53f/f;KRas;Apln-/y Ctrl vs. p53f/f;KRas;Apln-/y Sut	0.0002
	p53f/f;KRas;Apln+/y Sut vs. p53f/f;KRas;Apln-/y Sut	<0.0001
