# Supplementary material for: Apelin inhibition prevents resistance and metastasis associated with anti‐angiogenic therapy
Source: EMBO Mol Med. 2019 Jun 24;11(8):e9266. doi: 10.15252/emmm.201809266 (PMC6685079; doi:10.15252/emmm.201809266)
Supplement: Supplementary file 2 — Expanded View Figures PDF [file EMMM-11-e9266-s002.pdf]

Expanded View Figures

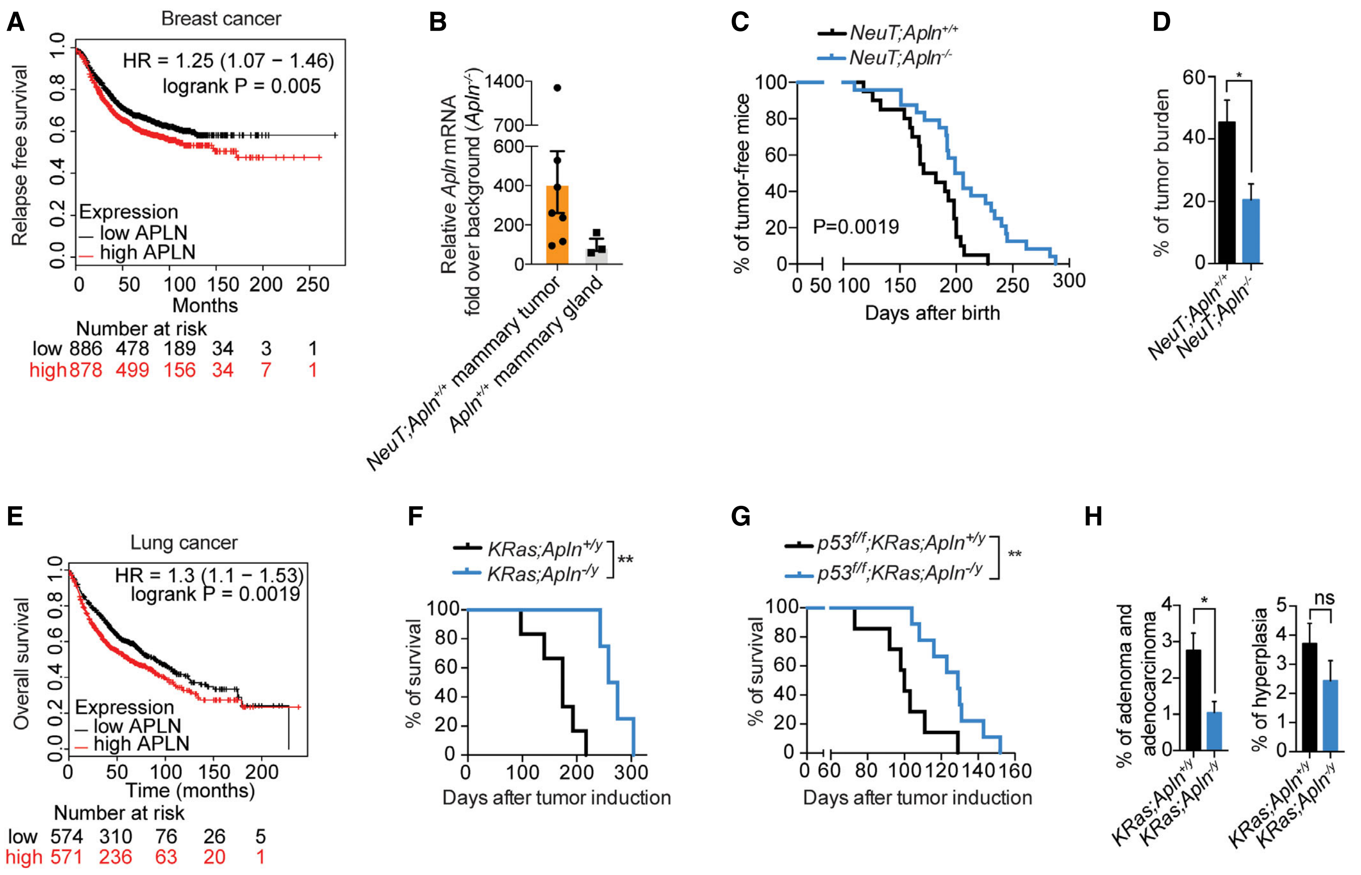

Figure EV1. Apelin inactivation improves survival and reduces tumor burden in mammary and lung cancer.

A Kaplan–Meier survival plot from the KM-plotter database (Győrffy *et al*, 2013) for high and low Apelin (APLN)-expressing groups in human breast cancer. Relapse-free survival. Patients were split by the median. Affymetrix Apelin ID 222856_at.

B RT-qPCR of Apelin (*Apln*) expression in *NeuT;Apln*$^{+/+}$ tumors (orange bar, $n = 7$) and purified normal (*Apln*$^{+/+}$) mammary gland epithelial cells (gray bar, $n = 3$). Data points from individual tumors or mammary glands and means (black bars) are shown.

C Kaplan–Meier plot for mammary tumor onset in *NeuT;Apln*$^{+/+}$ ($n = 20$) and *NeuT;Apln*$^{-/-}$ ($n = 24$) mice. $P = 0.0019$; log-rank test.

D Mean percentages ± SEM of tumor burden in *NeuT;Apln*$^{+/+}$ ($n = 6$) and *NeuT;Apln*$^{-/-}$ ($n = 9$) mammary glands assessed 4 weeks after tumor onset. $^*P < 0.05$; *t*-test.

E Kaplan–Meier plot from the KM-plotter database (Győrffy *et al*, 2013) for high and low APELIN-expressing groups in lung cancer patients. Overall survival. Patients were split by the median. Affymetrix Apelin ID 222856_at.

F Kaplan–Meier plot for survival in *KRas;Apln*$^{+/y}$ ($n = 6$) and *KRas;Apln*$^{-/y}$ ($n = 4$) mice with non-small cell lung cancer (NSCLC) after oncogenic KRas induction by AdenoCre inhalation. $^{**}P < 0.01$; log-rank test.

G Kaplan–Meier plot for survival in *p53*$^{f/f}$*;KRas;Apln*$^{+/y}$ ($n = 7$) and *p53*$^{f/f}$*;KRas;Apln*$^{-/y}$ ($n = 9$) mice with NSCLC after AdenoCre inhalation. $^{**}P < 0.01$; log-rank test.

H Percentages and representative histology of lung adenoma/adenocarcinoma and hyperplasia (±SEM) in age-matched *KRas;Apln*$^{+/y}$ ($n = 4$) and *KRas;Apln*$^{-/y}$ ($n = 4$) mice analyzed 18 weeks after AdenoCre inhalation. $^*P < 0.05$, ns = not significant; *t*-test; three sections per lung were analyzed.

Source data are available online for this figure.

© 2019 The Authors

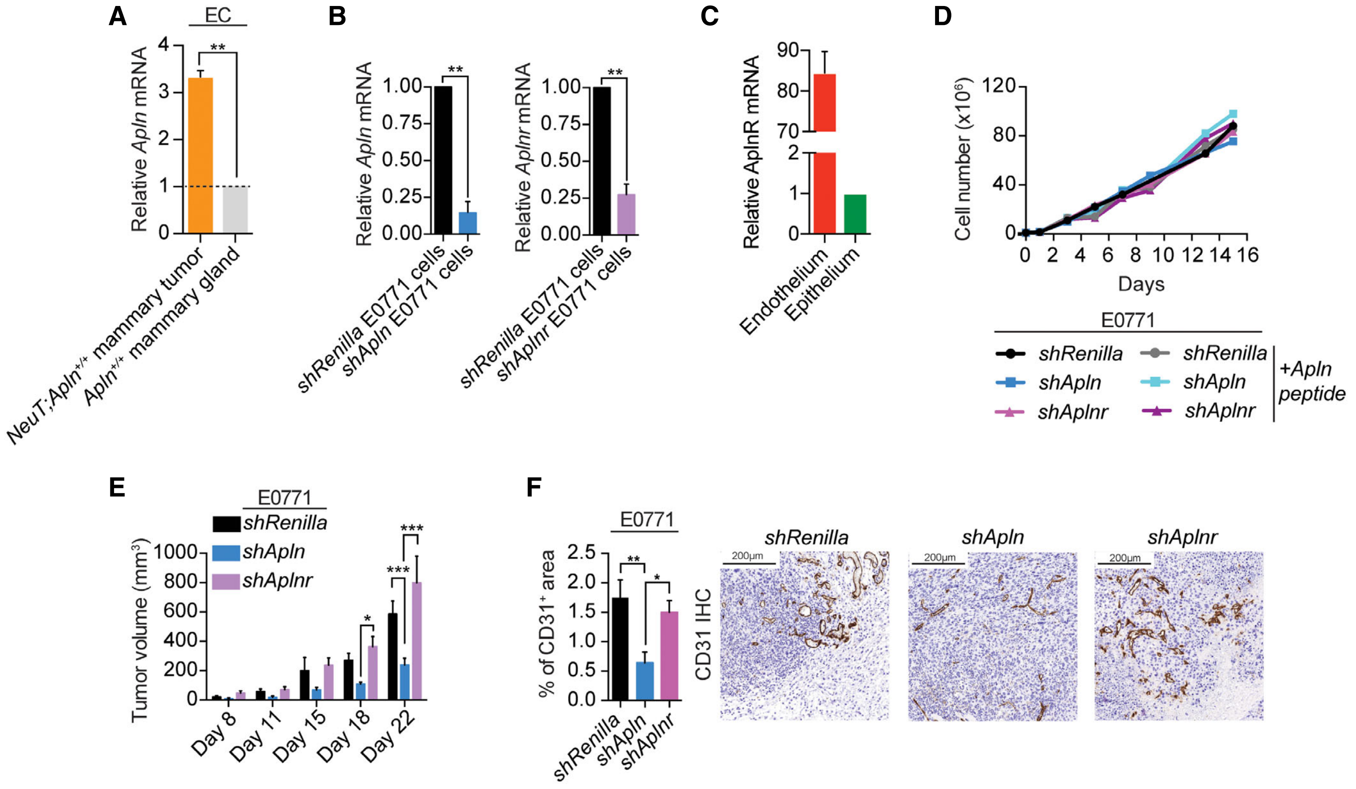

Figure EV2. Tumor cell-derived Apelin induces angiogenesis in a paracrine manner.

A RT-qPCR of Apelin *(Apln)* expression in endothelial cells (ECs) isolated from *NeuT;Apln*$^{+/+}$ tumors, normal *Apln*$^{+/+}$ mammary glands. Data are shown as relative mRNA levels normalized to the normal mammary gland endothelium (set to 1) ± SEM. n = 2 per cohort; **$P < 0.01$, t-test.

B RT-qPCR of *Apln* and *Aplnr* in control E0771 mammary cancer cells (*shRenilla*) and Apelin or Apelin receptor-depleted E0771 cells (*shApln* and *shAplnr*, respectively). Data are shown as relative mRNA levels normalized to control E0771 cells (set to 1) ± SEM. n = 2 per cohort; **$P < 0.01$; t-test.

C Relative levels of AplnR mRNA (mean ± SEM) in isolated tumor endothelial cells compared to tumor epithelial cells from NeuT-driven *Apln*$^{+/+}$ mouse mammary tumors; n = 4 per cohort. Data are shown as relative mRNA levels normalized to epithelial cells (set to 1) ± SEM.

D *In vitro* growth curves of *shRenilla*, *shApln* or *shAplnr* E0771 cells in the absence or presence of an active Apelin peptide (AplnPyr13, 1000 nM). No difference in growth was observed. A representative experiment is shown.

E Tumor volume, followed over time, of orthotopically injected *shRenilla*, *shApln,* and *shAplnr* E0771 cells. Data were determined using calipers and are shown as mean tumor volumes ± SEM. n = 4 syngeneic C57BL/6J mice per cohort; *$P < 0.05$, ***$P < 0.001$, two-way ANOVA.

F Quantification (mean percentages ± SEM) and representative immunohistochemistry of CD31^{+} area in *shRenilla* (n = 6), *shApln* (n = 6) or *shAplnr* (n = 7) E0771 mammary tumors in C57BL/6J wild-type mice, analyzed on day 12 post-orthotopic injection. *$P < 0.05$, **$P < 0.01$; one-way ANOVA. Scale bars = 200 µm.

Figure EV3. Gene expression analysis in Apelin-depleted tumor endothelial cells and endothelial sprouts.

A Ingenuity pathway analysis for upstream regulators predicted to be inhibited based on significantly differentially expressed genes from RNA-Seq transcriptome analysis of $CD31^+$/$CD105^+$ endothelial cells sorted from tumors established by E0771 *shRenilla* (n = 6) or *shApln* (n = 3) cells orthotopically injected into C57BL/6J *Apln*$^{+/+}$ or *Apln*$^{-/-}$ mice, respectively. Tumors were harvested day 25 post-injection.

B Representative immunofluorescence images of sprouting embryoid bodies (EBs) derived from CCE mouse embryonic stem cells (mESCs) after 6 days of sprouting initiation. EBs were treated with VEGF (30 ng/ml) only or VEGF (30 ng/ml) followed by AplnPyr13 (1,000 nM) upon sprouting initiation. Scale bars = 200 μm.

C Apelin knockout strategy and reversion of knockout by Cre addition in haploid murine embryonic stem cells (mESCs). If in sense, integration of the splice acceptor into an intron results in gene disruption due to splicing (STOP); after reverting the cassette into antisense by Cre addition, the cassette is spliced out and gene transcription occurs from the "repaired" locus (GO). SA: splicing acceptor; pA: polyA.

D RT-qPCR for Apelin in mESCs with *Apln* STOP and *Apln* GO integrations of the splice acceptor as shown in Appendix Fig S3C.

E Growth curve of *Apln* GO mESCs and their genetically repaired sister mESCs (*Apln* STOP). A representative experiment is shown.

F Percentages (mean ± SEM) of $CD31^+$ ECs evaluated by FACS from mosaic EBs formed from mESCs of 1:1 mixed Apelin GO ($mCherry^+Cre^+$): Apelin STOP (GFP^+). ***$P < 0.001$, Mann–Whitney test; n = 4 per cohort. Representative FACS analysis dot plot is shown.

G Ingenuity pathway analysis (IPA) predicting inhibited upstream regulators based on differentially expressed genes in RNA-Seq analysis from $CD31^+$ endothelial cells (ECs) isolated from sprouting EBs from either repaired *Apln* GO cells stimulated with VEGF (30 ng/ml) and Apelin (1,000 nM; full presence of Apelin) or *Apln* STOP sister cells stimulated with VEGF and control DMSO (total absence of Apelin).

H Heatmap of the genes downstream of VEGF (Appendix Fig S3F) comparing differentially expressed genes in RNA-Seq analysis from $CD31^+$ endothelial cells isolated from sprouting EBs from either repaired *Apln* GO mESCs stimulated with VEGF (30 ng/ml) and Apelin (1,000 nM; +*Apln*) or *Apln* STOP sister cells stimulated with VEGF and control DMSO (−*Apln*).

 © 2019 The Authors

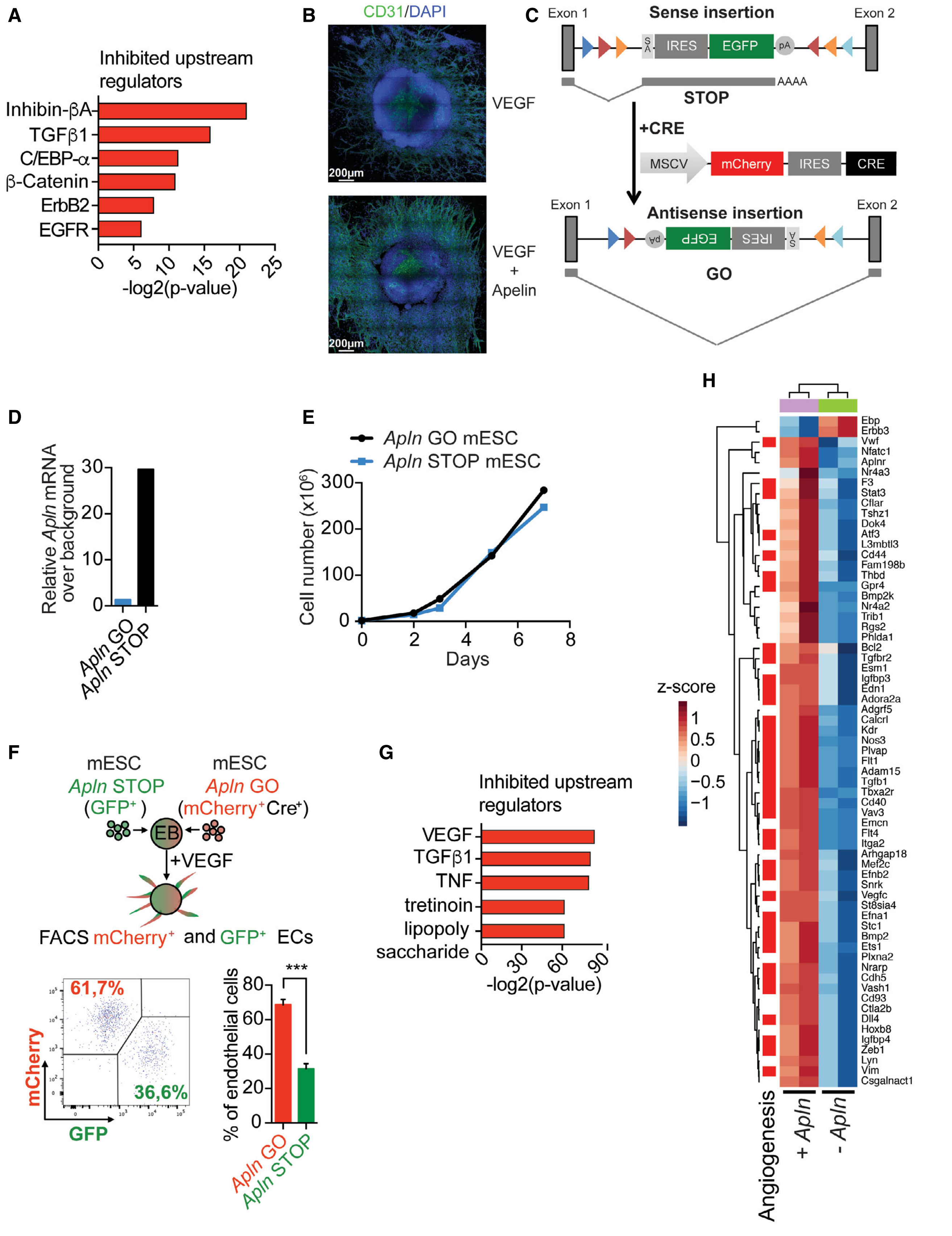

A
Inhibited upstream regulators
Inhibin-βA
TGFβ1
C/EBP-α
β-Catenin
ErbB2
EGFR
0 5 10 15 20 25
-log2(p-value)
B
CD31/DAPI
VEGF
VEGF + Apelin
200µm
C
Exon 1
Sense insertion
Exon 2
SA
IRES
EGFP
pA
AAAA
STOP
+CRE
MSCV
mCherry
IRES
CRE
Antisense insertion
GO
D
Relative Apln mRNA over background
30
20
10
0
Apln GO
Apln STOP
E
Apln GO mESC
Apln STOP mESC
Cell number (x10^6)
300
200
100
0
0 2 4 6 8
Days
F
mESC
Apln STOP
(GFP+)
mESC
Apln GO
(mCherry+Cre+)
EB
+VEGF
FACS mCherry+ and GFP+ ECs
61,7%
36,6%
mCherry
GFP
% of endothelial cells
80
60
40
20
0
***
Apln GO
Apln STOP
G
Inhibited upstream regulators
VEGF
TGFβ1
TNF
tretinoin
lipopoly saccharide
0 30 60 90
-log2(p-value)
H
z-score
1
0.5
0
−0.5
−1
Ebp
Erbb3
Vwf
Nfatc1
Aplnr
Nr4a3
F3
Stat3
Cflar
Tshz1
Dok4
Atf3
L3mbtl3
Cd44
Fam198b
Thbd
Gpr4
Bmp2k
Nr4a2
Trib1
Rgs2
Phlda1
Bcl2
Tgfbr2
Esm1
Igfbp3
Edn1
Adora2a
Adgrf5
Calcrl
Kdr
Nos3
Plvap
Flt1
Adam15
Tgfb1
Tbxa2r
Cd40
Vav3
Emcn
Flt4
Itga2
Arhgap18
Mef2c
Efnb2
Snrk
Vegfc
St8sia4
Efna1
Stc1
Bmp2
Ets1
Plxna2
Nrarp
Cdh5
Vash1
Cd93
Ctla2b
Dll4
Hoxb8
Igfbp4
Zeb1
Lyn
Vim
Csgalnact1
Angiogenesis
+ Apln
- Apln

Figure EV3.

Figure EV4. Combining Apelin depletion and current anti-angiogenic treatment reduces mammary tumor growth *in vivo*.

A Volume of mammary tumors from control (*shRenilla*) and Apelin-depleted (*shApln*) E0771 cells orthotopically injected in C57BL/6J *Apln*$^{+/+}$ or *Apln*$^{-/-}$ mice, respectively, as indicated. E0771-injected mice were left untreated (control) or *in vivo* treated with sunitinib (60 mg/ml, five times a week) starting on day 5 after cell injection. Tumor volumes were determined on the indicated time points using calipers and are shown as mean tumor volumes ± SEM. *Apln*$^{+/+}$;*shRenilla* control (n = 3), *Apln*$^{-/-}$;*shApln* control (n = 3), *Apln*$^{+/+}$;*shRenilla* sunitinib (n = 3), *Apln*$^{-/-}$;*shApln* sunitinib (n = 5); *$P < 0.05$, **$P < 0.01$, ***$P < 0.001$, two-way ANOVA.

B Tumor volumes, followed over the indicated time, of orthotopically injected E0771 *shRenilla* or *shApln* cells in C57BL/6J mice left untreated (control) or treated daily from day 10 after tumor injection with axitinib (30 mg/kg). Tumor volumes were measured using calipers and are shown as mean tumor volumes ± SEM. The mice that rejected the tumor were excluded from the analysis. *Apln*$^{+/+}$;*shRenilla* control (n = 5), *Apln*$^{-/-}$;*shApln* control (n = 3), *Apln*$^{+/+}$;*shRenilla* Axitinib (n = 3), *Apln*$^{-/-}$;*shApln* Axitinib (n = 3); *$P < 0.05$, **$P < 0.01$, ***$P < 0.001$, two-way ANOVA.

C Tumor volumes, followed over the indicated time, of orthotopically injected E0771 *shRenilla* or *shApln* cells in C57BL/6J mice and treated with an anti-VEGFR2 antibody or its isotype control (control) from day 10 after tumor injection (1 mg per mouse, twice a week). Tumor volumes were measured using calipers and are shown as mean tumor volumes ± SEM. n = 5 mice per cohort; **$P < 0.01$, ***$P < 0.001$, two-way ANOVA.

D Ki67 quantification of large size-matched mammary tumors from *NeuT;Apln*$^{+/+}$ and *NeuT;Apln*$^{-/-}$ mice, left untreated (control) or treated with sunitinib (60 mg/kg, starting at tumor onset, three times per week). Data show percentages of Ki67^{+} cells per breast tumor ± SEM. *NeuT;Apln*$^{+/+}$ control (n = 5), *NeuT;Apln*$^{-/-}$ control (n = 7), *NeuT;Apln*$^{+/+}$ sunitinib (n = 5), *NeuT;Apln*$^{-/-}$ sunitinib (n = 4); **$P < 0.01$; t-test. Right panels show representative Ki67 intra-tumoral stainings. Scale bars = 200 μm (large panels) and 50 μm (insets).

 © 2019 The Authors

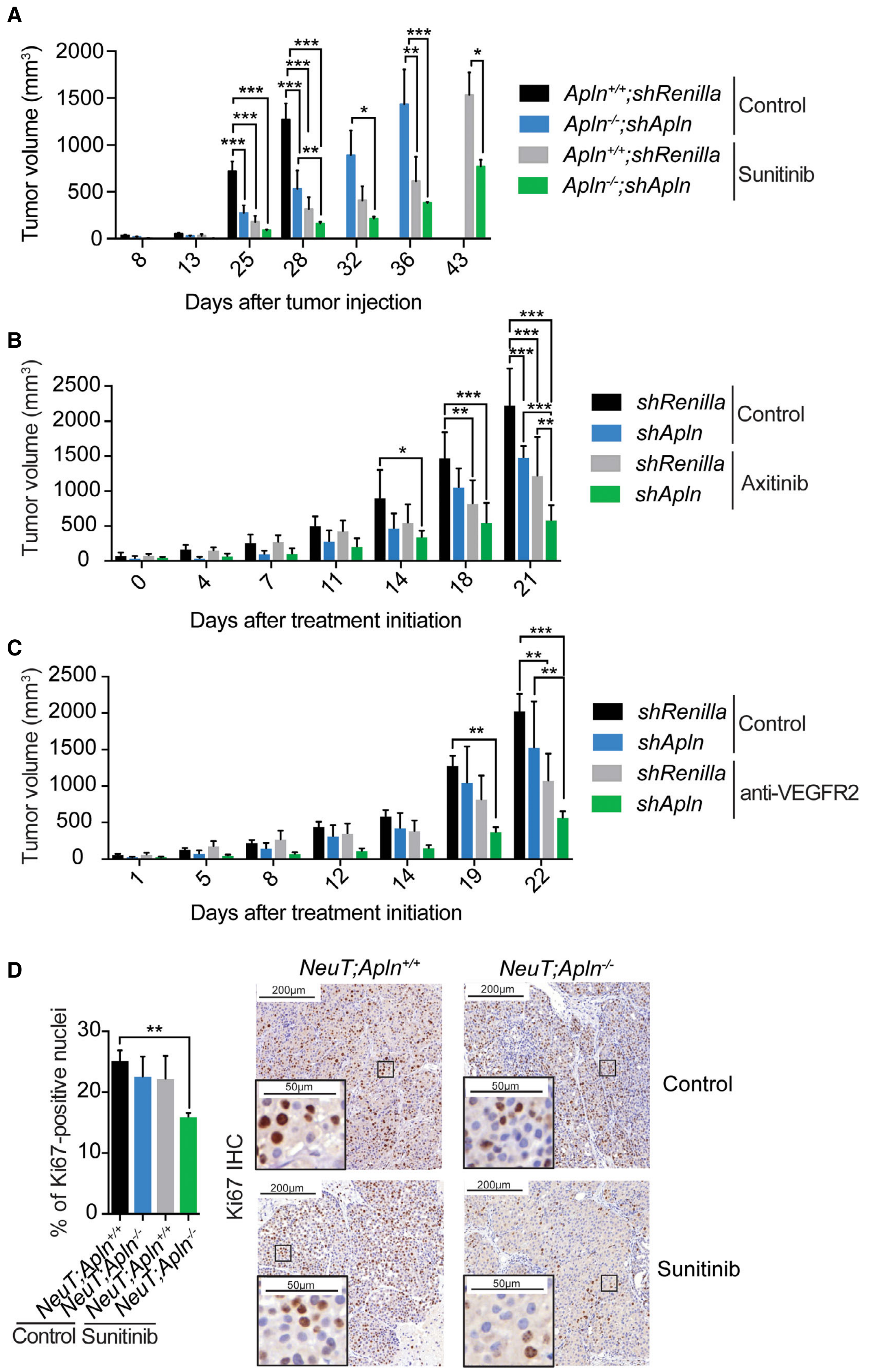
A
Tumor volume (mm3)
Days after tumor injection
Apln+/+;shRenilla
Apln-/-;shApln
Apln+/+;shRenilla
Apln-/-;shApln
Control
Sunitinib
B
Tumor volume (mm3)
Days after treatment initiation
shRenilla
shApln
shRenilla
shApln
Control
Axitinib
C
Tumor volume (mm3)
Days after treatment initiation
shRenilla
shApln
shRenilla
shApln
Control
anti-VEGFR2
D
% of Ki67-positive nuclei
NeuT;Apln+/+
NeuT;Apln-/-
NeuT;Apln+/+
NeuT;Apln-/-
Control
Sunitinib
Ki67 IHC
NeuT;Apln+/+
NeuT;Apln-/-
200μm
50μm
Control
Sunitinib

Figure EV4.

Figure EV5. Apelin inhibition protects from anti-angiogenic therapy-induced metastases.

A Number of metastatic lung foci in untreated (control) and sunitinib-treated (60 mg/kg, three times a week from tumor initiation) *NeuT;Apln*$^{+/+}$ and *NeuT;Apln*$^{-/-}$ mice with large size-matched tumors. Data of individual lung sections and means (black bars) are shown. Identified outliers by ROUT method (Q = 1%) were obviated. Right panels show representative H&E images, where black arrows and insets indicate metastatic foci. Scale bars = 1,000 μm (large panels) and 50 μm (insets). $^{***}P < 0.001$; one-way ANOVA; *NeuT;Apln*$^{+/+}$ control (n = 9), *NeuT;Apln*$^{-/-}$ control (n = 8), *NeuT;Apln*$^{+/+}$ sunitinib (n = 6), *NeuT;Apln*$^{-/-}$ sunitinib (n = 7); and three sections per lung were analyzed.

B, C Number of metastatic lung foci per lung section from 1 to 1.5 cm^3 size-matched E0771 control (*shRenilla*) and Apelin-depleted (*shApln*) E0771 tumors orthotopically injected into C57BL/6J *Apln*$^{+/+}$ (B) or orthotopically injected into C57BL/6J *Apln*$^{+/+}$ or *Apln*$^{-/-}$ mice (C). Mice were either left untreated (control) or treated with sunitinib (60 mg/kg, starting at day 5 after tumor injection, five times a week). Data of individual lung sections and means (black bars) are shown. Right panels show representative H&E images, where black arrows and insets indicate metastatic foci. Scale bars = 1,000 μm (large panels) and 50 μm (insets). E0771 *shRenilla* control (n = 7), E0771 *shApln* control (n = 4), E0771 *shRenilla* sunitinib (n = 5), E0771 *shApln* sunitinib (n = 3) (B) or *Apln*$^{+/+}$ *shRenilla* control (n = 3), *Apln*$^{-/-}$ *shApln* control (n = 3), *Apln*$^{+/+}$ *shRenilla* sunitinib (n = 2), *Apln*$^{-/-}$ *shApln* sunitinib (n = 3) (C) and three sections per lung were analyzed. $^{**}P < 0.01$; $^{***}P < 0.001$; one-way ANOVA.

© 2019 The Authors

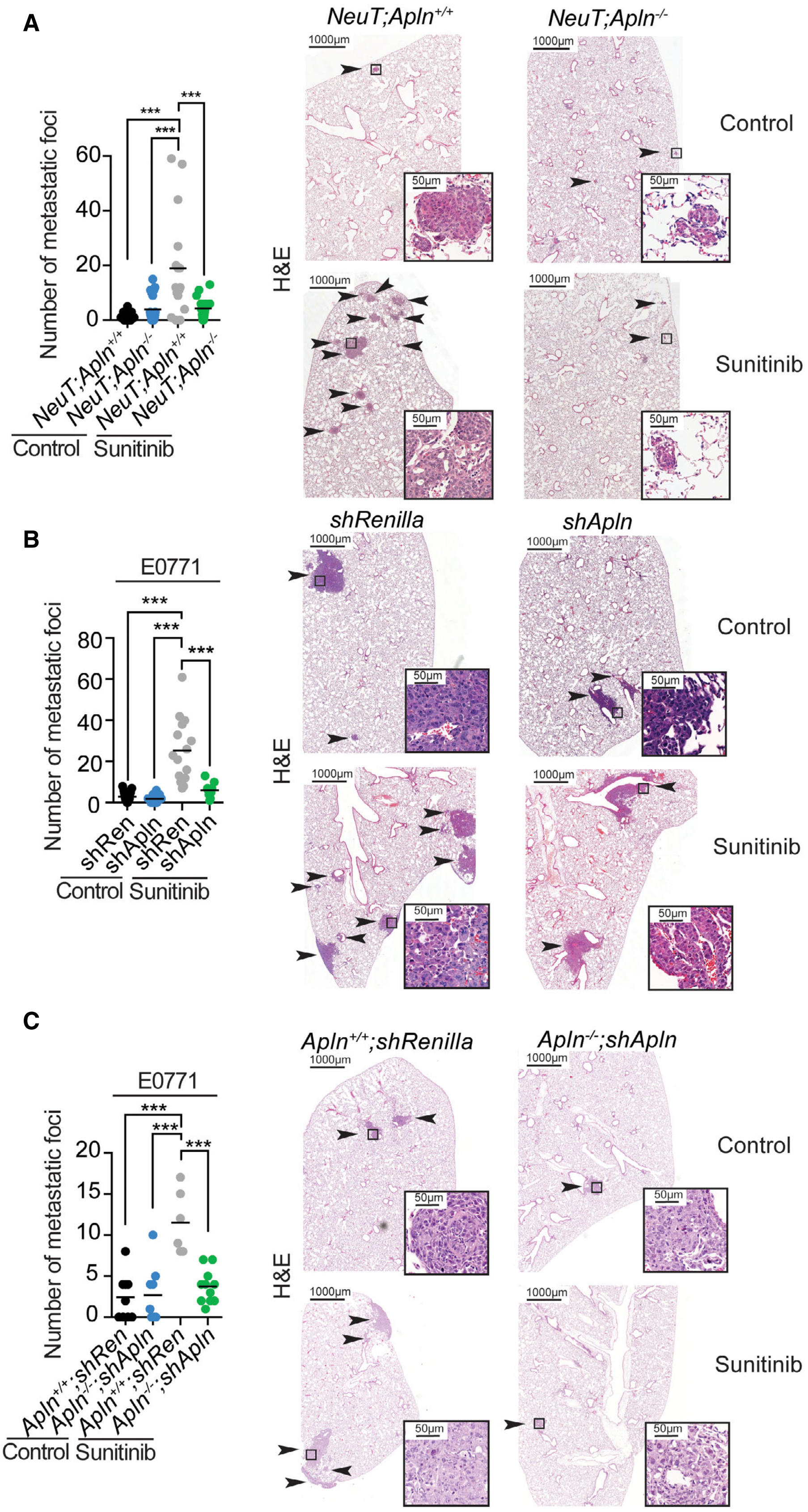
A
NeuT;Apln+/+
NeuT;Apln-/-
Control
Sunitinib
H&E
Number of metastatic foci
NeuT;Apln+/+
NeuT;Apln-/-
NeuT;Apln+/+
NeuT;Apln-/-
B
E0771
shRenilla
shApln
shRen
shApln
shRen
shApln
C
Apln+/+;shRenilla
Apln-/-;shApln
Apln+/+;shRen
Apln-/-;shApln
Apln+/+;shRen
Apln-/-;shApln
1000µm
50µm

Figure EV5.
