# Supplementary material for: Apelin inhibition prevents resistance and metastasis associated with anti‐angiogenic therapy
Source: EMBO Mol Med. 2019 Jun 24;11(8):e9266. doi: 10.15252/emmm.201809266 (PMC6685079; doi:10.15252/emmm.201809266)
Supplement: Supplementary file 4 — Source Data for Expanded View and Appendix [file EMMM-11-e9266-s007.zip › EMM-09266-sd/EMM-2018-09266-V4_Source_FigS2.pdf]

Appendix Figure S2A			
Survival after tumor induction [d]			
p53$^{f/f}$;KRas;Apln$^{+/y}$	p53$^{f/f}$;KRas;Apln$^{-/y}$	p53$^{f/f}$;KRas;Apln$^{+/y}$ + Sunitinib	p53$^{f/f}$;KRas;Apln$^{-/y}$ + Sunitinib
98	129	121	138
129	108	115	125
103	104	128	179
100	131	125	193
111	143	139	167
73	123	135	137
92	130	145	146
121	152	178	146
111	116	156	161
103	121	122	154
115	142	132	
	128	146	
