# Supplementary material for: Apelin inhibition prevents resistance and metastasis associated with anti‐angiogenic therapy
Source: EMBO Mol Med. 2019 Jun 24;11(8):e9266. doi: 10.15252/emmm.201809266 (PMC6685079; doi:10.15252/emmm.201809266)
Supplement: Supplementary file 4 — Source Data for Expanded View and Appendix [file EMMM-11-e9266-s007.zip › EMM-09266-sd/EMM-2018-09266-V4_Source_FigEV1.pdf]

Figure EV1C	
Survival after birth [d]	
NeuT;Apln$^{+/+}$	NeuT;Apln$^{-/-}$
228	192
204	288
171	165
200	283
133	234
182	151
161	199
118	226
159	240
154	151
126	172
193	245
200	231
168	213
207	185
190	110
168	192
167	199
198	262
198	193
	191
	206
	206
	244

Figure EV1F	
Survival after tumor induction [d]	
Kras;Apln$^{+/y}$	Kras;Apln$^{-/y}$
97	243
140	258
174	275
174	304
193	
217	

Figure EV1G	
Survival after tumor induction [d]	
p53$^{f/f}$;KRas;Apln$^{+/y}$	p53$^{f/f}$;KRas;Apln$^{-/y}$
98	129
129	108
103	104
100	131
111	143
73	123
92	130
	152
	116
