# Supplementary material for: Apelin inhibition prevents resistance and metastasis associated with anti‐angiogenic therapy
Source: EMBO Mol Med. 2019 Jun 24;11(8):e9266. doi: 10.15252/emmm.201809266 (PMC6685079; doi:10.15252/emmm.201809266)
Supplement: Supplementary file 6 — Source Data for Figure 1 [file EMMM-11-e9266-s004.pdf]

Figure 1A	
Survival after tumor onset [d]	
NeuT;Apln$^{+/+}$	NeuT;Apln$^{-/-}$
40	70
70	69
80	59
54	65
54	85
60	55
30	65
66	85
33	65
43	91
46	
