# Supplementary material for: Apelin inhibition prevents resistance and metastasis associated with anti‐angiogenic therapy
Source: EMBO Mol Med. 2019 Jun 24;11(8):e9266. doi: 10.15252/emmm.201809266 (PMC6685079; doi:10.15252/emmm.201809266)
Supplement: Supplementary file 7 — Source Data for Figure 3 [file EMMM-11-e9266-s005.pdf]

Figure 3A			
Survival after tumor onset [d]			
NeuT;Apln$^{+/+}$ + Sunitinib	NeuT;Apln$^{-/-}$ + Sunitinib	NeuT;Apln$^{+/+}$	NeuT;Apln$^{-/-}$
69	163	46	41
55	69	38	55
71	110	50	55
41	57	23	132
94	120	82	45
68	89	71	86
55	113	33	65
55	101	38	80
117	50		101
117	179		99
110	131		81
	128		
