# Supplementary material for: Apelin inhibition prevents resistance and metastasis associated with anti‐angiogenic therapy
Source: EMBO Mol Med. 2019 Jun 24;11(8):e9266. doi: 10.15252/emmm.201809266 (PMC6685079; doi:10.15252/emmm.201809266)
Supplement: Supplementary file 8 — Source Data for Figure 6 [file EMMM-11-e9266-s006.pdf]

Figure 6c	
Progression-free survival after sunitinib treatment	
High APLN	Low APLN
102	709
570	463
133	143
82	379
629	203
572	1140
162	540
462	691
338	708
706	132
172	240
460	391
236	222
748	623
280	456
158	623
450	660
138	331
343	846
799	350
383	120
160	534
37	154
83	185
273	674
116	1257
525	288
	793

Figure 6d			
Progression-free survival after sunitinib treatment			
Low APLN/ Low VEGF	Low APLN/ High VEGF	High APLN/ Low VEGF	High APLN/ High VEGF
222	143	450	162
456	288	160	460
240	674	280	158
534	379	338	82
623	793	383	748
691	708	138	102
1257	203	116	37
1140	350	572	172
540	120	525	570
846	185	343	133
660	463	273	799
623	391	462	236
	331	83	
	132	629	
	154	706	
	709		
