# Supplementary material for: Apelin inhibition prevents resistance and metastasis associated with anti‐angiogenic therapy
Source: EMBO Mol Med. 2019 Jun 24;11(8):e9266. doi: 10.15252/emmm.201809266 (PMC6685079; doi:10.15252/emmm.201809266)
Supplement: Supplementary file 3 — Movie EV1 [file EMMM-11-e9266-s003.zip › EMM201809266V3_MovieEV1/Movie_EV1.doc]

**Movie EV1 - 3D reconstruction of representative mammary tumors** (in pink) analysed by MRI from untreated (control) and sunitinib-treated *NeuT;Apln^+/+^* and *NeuT;Apln^-/-^* mice (60mg/kg, three times per week), assessed 6 weeks after mammary tumors were size-matched.
